# Pancreatic ductal adenocarcinoma with ITSN1-ALK fusion: sustained response to alectinib with 19-month progression-free survival

**DOI:** 10.1093/oncolo/oyag209

**Published:** 2026-05-27

**Authors:** Chaoxing Liu, Xiaoyan Wang, Lixuan Chen

**Affiliations:** Department of Radiation Oncology, Shijiazhuang People’s Hospital, Shijiazhuang, Hebei Province, China; Department of Radiation Oncology, Shijiazhuang People’s Hospital, Shijiazhuang, Hebei Province, China; Department of Radiation Oncology, Shijiazhuang People’s Hospital, Shijiazhuang, Hebei Province, China

**Keywords:** pancreatic ductal adenocarcinoma, ALK fusion, ITSN1-ALK, targeted therapy, precision oncology

## Abstract

**Background:**

Pancreatic ductal adenocarcinoma (PDAC) harboring ALK fusions is exceptionally rare, with limited data on clinical outcomes with ALK inhibitor therapy.

**Case summary:**

A 70-year-old man presented with stage IV PDAC (pancreatic tail primary with liver and lymph node metastases). Following progression on first-line gemcitabine/nab-paclitaxel, tumor next-generation sequencing identified an ITSN1-ALK fusion (62.12% abundance) and TP53 mutation. The patient received alectinib 600 mg twice daily beginning at Month 4. Tumor markers declined markedly (CA19-9 from 234 to 12.7 U/mL by Month 7), and radiographic stability was maintained through Month 22. Upon progression at Month 23, treatment was switched to lorlatinib followed by capecitabine plus nimotuzumab. The patient remains alive at last follow-up (Month 29). Progression-free survival on alectinib was 19 months—substantially exceeding the median of 5.0 months reported for ALK fusion-positive gastrointestinal cancers.

**Conclusion:**

This first report of ITSN1-ALK fusion in PDAC demonstrates that ALK inhibition can produce durable, clinically meaningful benefit. This case reinforces the importance of comprehensive genomic profiling in KRAS-wildtype PDAC to identify actionable rare fusions.

**Implications for practice:**

Oncologists should consider broad next-generation sequencing, including fusion detection, for patients with KRAS-wildtype PDAC. When an ALK fusion is identified, ALK inhibitor therapy can yield durable disease control.

## Graphical Abstract

**Graphical Abstract:**
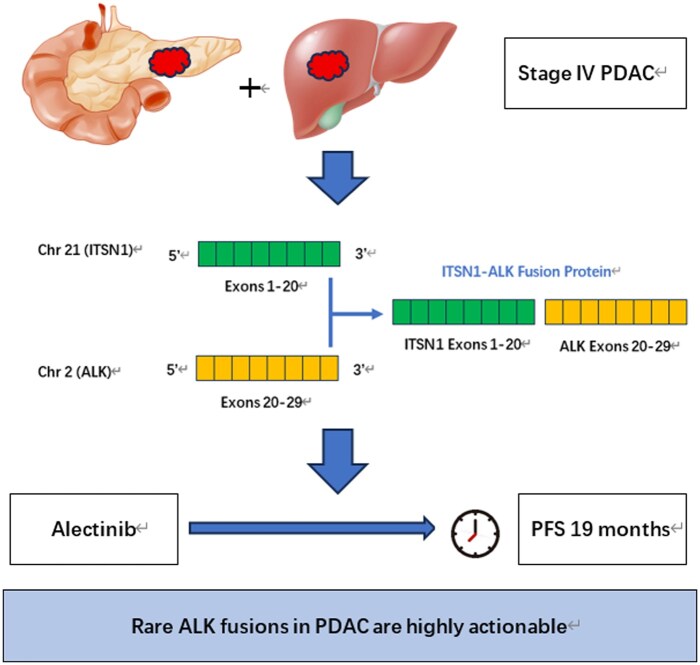

Key Points

- **Clinical context:** Pancreatic ductal adenocarcinoma (PDAC) is rarely associated with targetable gene fusions. Comprehensive genomic profiling of KRAS-wildtype tumors can identify rare drivers.
- **Key findings:** This is the first report of an ITSN1-ALK fusion in PDAC. Treatment with the ALK inhibitor alectinib produced a sustained biochemical response and 19 months of progression-free survival—substantially longer than the median 5.0 months reported for ALK fusion-positive gastrointestinal cancers.
- **Implications for practice:** Oncologists should consider broad next-generation sequencing, including fusion detection, for patients with KRAS-wildtype PDAC. When an ALK fusion is identified, ALK inhibitor therapy can yield durable disease control.

## Patient story

The patient is a 70-year-old retired factory worker who had been in good health until he developed persistent left-sided abdominal pain. He had no family history of cancer. After learning he had stage IV pancreatic cancer, he was worried about his prognosis but remained determined to pursue treatment. He initially received standard chemotherapy, which caused fatigue and mild decreases in his blood counts. When his cancer progressed, he underwent genetic testing that revealed a rare abnormality. He then began a targeted therapy (alectinib) that he took as pills at home. Within weeks, his pain resolved, and his tumor markers dropped to normal. He was able to return to his daily activities, including walking and spending time with his family, for nearly 19 months before his cancer eventually progressed. He continues to receive treatment and remains alive more than 2 years after his diagnosis. The patient has given written consent to share his story.

## Molecular tumor board (MTB)

### Case presentation

A 70-year-old man (ECOG 2) presented with left upper quadrant pain. Imaging showed a pancreatic tail mass with liver metastases and retroperitoneal lymphadenopathy. Liver biopsy confirmed pancreatic adenocarcinoma. Tumor markers: CA19-9 234 U/mL, CA72-4 50.8 U/mL.

### First-line therapy

The patient received gemcitabine (1000 mg/m^2^, d1,8) plus nab-paclitaxel (125 mg/m^2^, d1) on a 21-day cycle (Months 1-5). Best response was stable disease (RECIST v1.1) with a slight decrease in the primary tumor. CA19-9 nadir was 42.9 U/mL at Month 2. By Month 4, CA19-9 rose to 170 U/mL and imaging showed progression.

### Genomic profiling

Formalin-fixed, paraffin-embedded (FFPE) liver biopsy tissue underwent targeted next-generation sequencing. An ITSN1-ALK fusion (exon 20: exon 20, abundance 62.12%) was identified, alongside a TP53 nonsense mutation. PD-L1 CPS was 5; microsatellite stable. KRAS was wildtype.

### Molecular rationale

ITSN1-ALK is a novel fusion partner. The retained ITSN1 coiled-coil domain promotes constitutive dimerization and activation of the ALK kinase domain, rendering it susceptible to ALK inhibitors.

### Second-line targeted therapy

At Month 6.0, alectinib 600 mg twice daily was initiated. CA19-9 decreased to 29.0 U/mL within 3 weeks and normalized (12.7 U/mL) by Month 7. Radiographic stable disease was maintained through Month 26. Progression-free survival on alectinib was 19 months (Months 7-26).

### Post-progression

Upon progression at Month 29, treatment was switched to lorlatinib, then capecitabine plus nimotuzumab. The patient remains alive at Month 31.

### Take-home message

This case highlights that rare ALK fusions in PDAC can be highly actionable, producing durable responses that exceed historical benchmarks for targeted therapy in gastrointestinal cancers.

## Patient update

The patient continued alectinib with excellent tolerance and no grade 3/4 adverse events. At Month 29, routine imaging revealed progression of hepatic metastases and new pulmonary nodules. Alectinib was discontinued. The patient subsequently received lorlatinib (Month 26-29) followed by capecitabine plus nimotuzumab (Month 29 onward). At the most recent follow-up (Month 31), the patient remains alive with ongoing supportive care. No new safety signals were observed. The patient has provided written consent for publication.

## Introduction

### Case report

#### Patient presentation

A 70-year-old man (ECOG performance status 2) presented with left upper quadrant abdominal pain. His medical history was unremarkable, with no family history of malignancy. Physical examination was non-revealing.

Abdominal computed tomography (CT) revealed a pancreatic tail mass with splenic hilar involvement, multiple hypodense liver lesions suspicious for metastases, and enlarged retroperitoneal lymph nodes. Ultrasound-guided liver biopsy demonstrated metastatic adenocarcinoma. Immunohistochemistry showed CK7(+), CK19(+), and CK8/18(+), consistent with pancreaticobiliary origin. The diagnosis was established as stage IV pancreatic tail adenocarcinoma (T3N1M1).

Baseline tumor markers were elevated: CA19-9 234 U/mL, CA72-4 50.8 U/mL, and CEA 5.93 ng/mL (Table 1). These initial studies were obtained at presentation (Month 0).

**Table 1.** Key tumor marker dynamics during treatment course.

Date	CA19-9 (U/mL)	CA72-4 (U/mL)	CEA (ng/mL)	Treatment phase
**Month 0**	234	50.8	5.93	Baseline (pre-chemotherapy)
**Month 3**	42.9	23.3	3.66	During chemotherapy (nadir)
**Month 5**	170	94.1	7.48	Pre-alectinib (progression)
**Month 7**	29.0	12.0	1.70	Alectinib (3 weeks)
**Month 10**	12.7	1.03	2.36	Alectinib (3 months)
**Month 13**	15.3	1.09	2.19	Alectinib (6 months)
**Month 16**	23.1	1.21	2.61	Alectinib (9 months)
**Month 22**	32.2	1.78	2.48	Alectinib (12 months)
**Month 25**	71.5	2.94	4.24	Alectinib (15 months)
**Month 29**	125	5.48	3.89	Progression (19 months)
**Month 31**	567	45.1	9.22	Post-progression

#### First-line chemotherapy

The patient received 8 cycles of gemcitabine (1000 mg/m^2^; absolute dose 1.6 g for body surface area 1.6 m^2^) and nab-paclitaxel (125 mg/m^2^; absolute dose 200 mg for BSA 1.6 m^2^) on a 21-day cycle from Month 1 to Month 5. The 21-day schedule was selected based on institutional practice and patient preference to reduce chemical toxicity; this regimen has been reported as an acceptable alternative to the standard 28-day MPACT schedule.^1^^,^^2^ Best response was stable disease according to RECIST v1.1, with a slight decrease in the primary tumor and some hepatic lesions on imaging at Month 2 (Table 1). CA19-9 nadir was 42.9 U/mL but subsequently rose. Treatment was well-tolerated with only mild cytopenias (grade 1 thrombocytopenia, grade 1 anemia).

By Month 5, CA19-9 had increased to 170 U/mL, and imaging showed progression of retroperitoneal lymphadenopathy with new left renal artery involvement. The patient declined second-line chemotherapy.

#### Genomic profiling

Formalin-fixed, paraffin-embedded liver biopsy tissue underwent targeted next-generation sequencing (NGS). This revealed an ITSN1-ALK gene fusion (abundance 62.12%), with breakpoints involving ITSN1 exon 20 and ALK exon 20 (confirmed by anchored multiplex PCR). A concurrent TP53 exon 6 nonsense mutation (abundance 51.47%) was identified. PD-L1 expression was CPS 5, and microsatellite status was stable. The tumor was KRAS wildtype.

#### ALK inhibitor therapy

**Figure 1.**
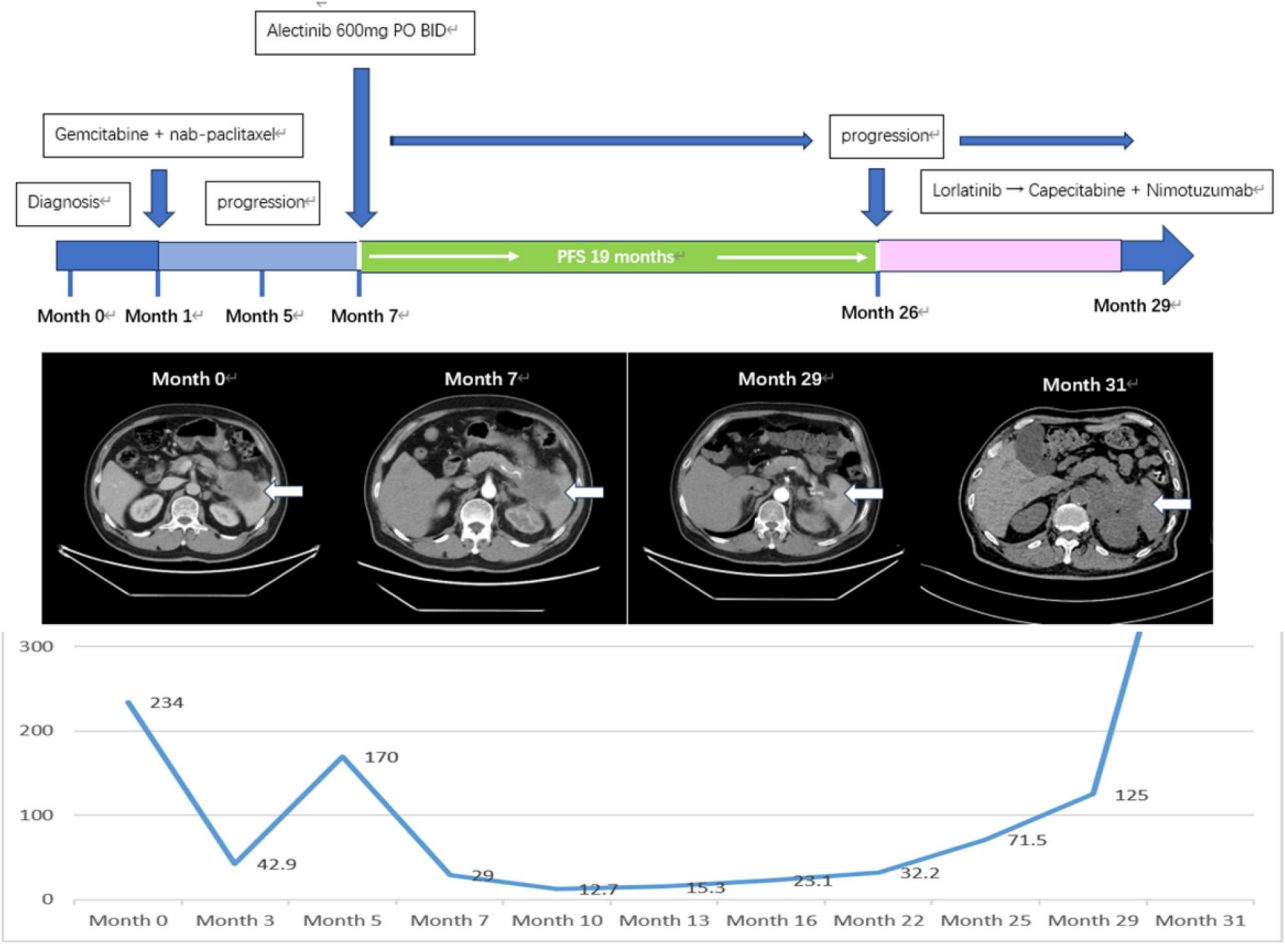
Clinical course and CA19-9 dynamics. Serial CA19-9 measurements (U/mL) from initial presentation (Month 0) through last follow-up (Month 31). The Y-axis uses a logarithmic scale. Treatment phases are indicated by colored shading: light blue for first-line chemotherapy (gemcitabine + nab-paclitaxel, Months 1-5); light green for alectinib therapy (Months 7-26); light pink for post-progression therapy (lorlatinib followed by capecitabine + nimotuzumab, Months 26-31). Key timepoints: Month 0 (CA19-9 234 U/mL); Month 3 (nadir during chemotherapy, 42.9 U/mL); Month 5 (progression, alectinib initiation, CA19-9 170 U/mL); Month 10 (biochemical normalization, 12.7 U/mL); Month 29 (radiographic progression, 125 U/mL). The 19-month progression-free survival on alectinib (Months 7-26) exceeds the median 5.0 months reported for ALK fusion-positive gastrointestinal cancers.^4^

According to the NGS results and the advice of MTB, at Month 6.5, the patient commenced alectinib 600 mg orally twice daily. Clinical and biochemical responses were marked. Within 3 weeks (Month 7), CA19-9 decreased to 29.0 U/mL, and by Month 10 had normalized to 12.7 U/mL (Figure 1). Tumor markers remained suppressed through Month 22.

Serial imaging confirmed partial response and sustained stable disease (RECIST v1.1), with no significant change in pancreatic and hepatic lesions on CT scans at Months 6, 9, 12, and 22. The patient remained asymptomatic and maintained excellent performance status (ECOG 0).

#### Durability of response

The patient continued alectinib with sustained disease control for 19 months (Month 7 through Month 26). At Month 29, rising CA19-9 (125 U/mL) prompted repeat imaging, which demonstrated progression of hepatic metastases and new pulmonary nodules (RECIST v1.1 progressive disease). Progression-free survival on alectinib was 19 months—substantially longer than the median 5.0 months reported for ALK fusion-positive gastrointestinal cancers.^3^

#### Subsequent therapy

Upon progression at Month 26, treatment was transitioned to lorlatinib. Subsequently, at Month 29, therapy was intensified with capecitabine (1.0 g twice daily days 1-14) plus nimotuzumab (400 mg intravenously day 1) on a 21-day cycle. As of the last follow-up at Month 31, the patient remains alive with overall survival exceeding 31 months from diagnosis.

## Discussion

This case report describes the first instance of ITSN1-ALK fusion in pancreatic ductal adenocarcinoma and documents an exceptional 19-month progression-free survival on alectinib (Table 2, Figure 1).

**Table 2.** Published Cases of ALK Fusion-Positive Pancreatic Tumors Treated with ALK Inhibitors

Reference	Histology	ALK Fusion Partner	ALKI	Best Response	PFS (months)
Ambrosini et al. 2022 [3]	PDAC (n=5)	Various	Various	2 PR, 2 SD	Median 5.0 (all GI)
Gower et al. 2020 [7]	PDAC	PPFIBP1-ALK	Alectinib → Lorlatinib	PR	NR (sequential response)
Ou et al. 2021 [8]	PDAC	EML4-ALK	Crizotinib → Alectinib	PR	8 (crizotinib)
Gaule et al. 2022 [9]	Acinar	KANK4-ALK	Alectinib	PR	NR
Shimada et al. 2017 [12]	PDAC	DCTN1-ALK	Preclinical	N/A	N/A
Meng F et al [13]	PDAC	KANK1-ALK	N/A	N/A	N/A
**Present case**	**PDAC**	**ITSN1-ALK**	**Alectinib → Lorlatinib**	**SD → PR**	**19**

Abbreviations: PDAC, pancreatic ductal adenocarcinoma; PR, partial response; SD, stable disease; NR, not reported; PFS, progression-free survival; ALKI, ALK inhibitor.

### Novel fusion partner: ITSN1-ALK

**Figure 2.**
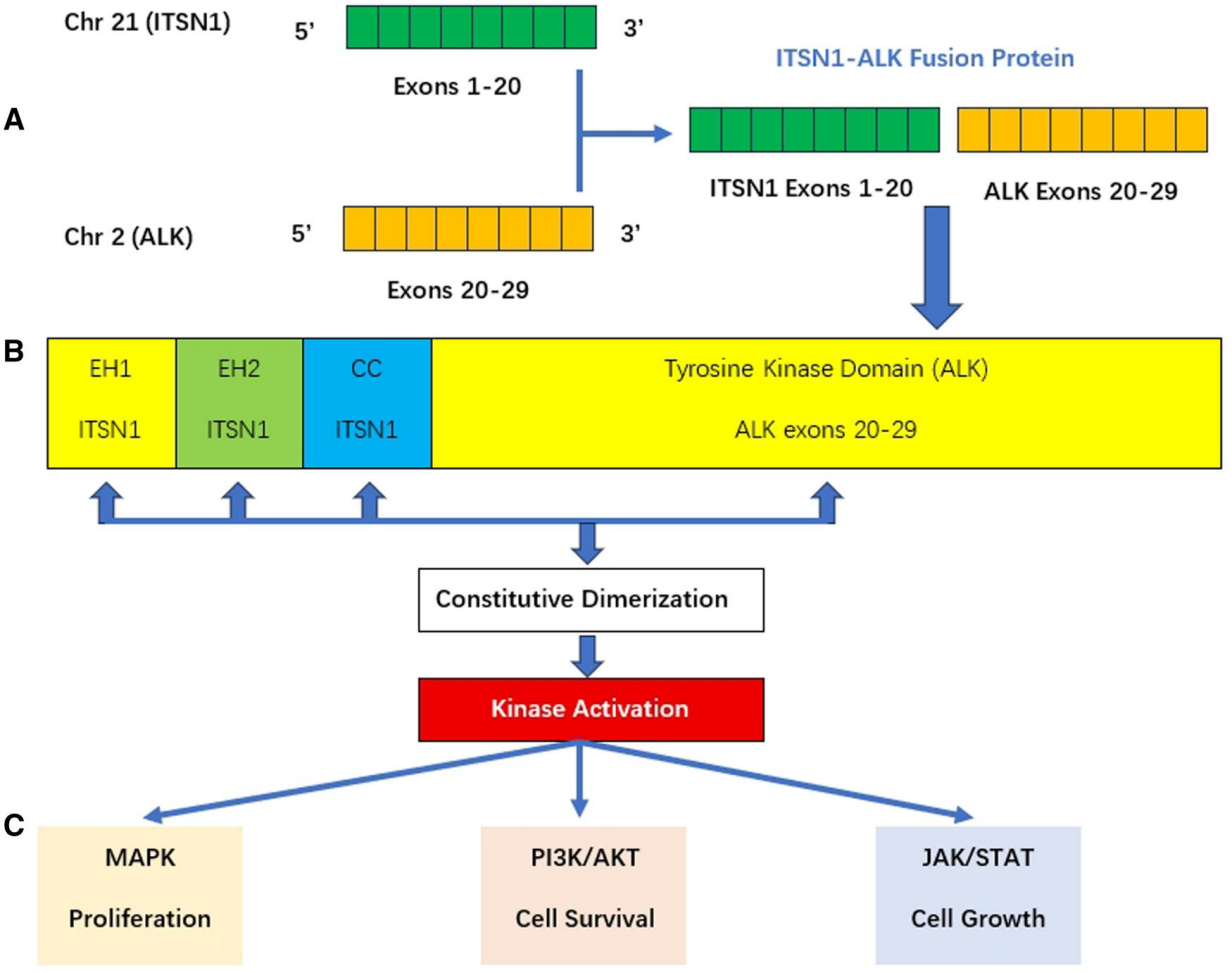
ITSN1-ALK fusion structure and functional mechanism. (A) Genomic rearrangement: fusion between ITSN1 (chromosome 21q22.2, exons 1-20) and ALK (chromosome 2p23.2, exons 20-29) at the breakpoint ITSN1 exon 20—ALK exon 20. (B) Domain architecture: the fusion retains the N-terminal ITSN1 domains (EH1, EH2, and coiled-coil [CC]) fused in-frame to the complete ALK tyrosine kinase domain. The CC domain mediates constitutive dimerization. (C) Functional consequence: constitutive dimerization leads to trans-autophosphorylation and activation of downstream signaling pathways (MAPK, PI3K/AKT, JAK/STAT), driving oncogenesis. The ALK kinase domain is directly targeted by alectinib.

ITSN1 (Intersectin 1) is a scaffold protein involved in endocytosis and signal transduction, located on chromosome 21q22.2. ITSN1-ALK fusions have been reported only rarely, with isolated cases in lung adenocarcinoma.^4^ The fusion identified in our patient involves ITSN1 exon 20 fused to ALK exon 20 (Figure 2A), retaining the complete ALK kinase domain while providing a dimerization domain from ITSN1 that promotes constitutive kinase activation—a mechanism analogous to other oncogenic ALK fusions^5^ (Figure 2B).

The high abundance (62.12%) suggests this fusion was a truncal driver event, consistent with the marked and sustained response to alectinib observed in our patient. The concurrent TP53 mutation did not appear to diminish ALK inhibitor sensitivity, aligning with observations in ALK-positive lung cancer where TP53 co-mutations affect prognosis but not initial targeted therapy response.^6^

### Exceptional durability of response

The 19-month PFS achieved in this case substantially exceeds previously reported outcomes. Ambrosini and colleagues^3^ reported a median PFS of only 5.0 months (95% CI, 3.68 to not reached) in their multicenter series of 13 ALK fusion-positive gastrointestinal malignancies, including 5 pancreatic primaries. Among those 5 patients, 2 achieved partial responses and 2 had stable disease, but durability varied widely.

Comparing our case to previously published PDAC cases with ALK fusions (Table 2), the 19-month PFS is notable. Gower and colleagues^7^ described a PPFIBP1-ALK fusion PDAC with response to alectinib and subsequent response to lorlatinib, though PFS duration on alectinib was not specified. Ou and colleagues^8^ reported an EML4-ALK fusion PDAC with response to crizotinib (PFS 8 months) followed by intracranial response to alectinib upon brain metastasis development. Gaule and colleagues^9^ described an ALK-KANK4 fusion in pancreatic acinar cell carcinoma with exceptional response to alectinib, though acinar histology differs biologically from conventional PDAC.

Several factors may explain the prolonged durability in our case: (1) high fusion abundance suggesting clonal dominance, (2) favorable fusion structure with intact kinase domain, (3) absence of concurrent resistance mechanisms, and (4) excellent drug exposure and adherence.

### Sequential ALK inhibition strategy

Following alectinib progression, our patient received lorlatinib, a third-generation ALK inhibitor with activity against most acquired resistance mutations. The feasibility of sequential ALK inhibition in PDAC was previously demonstrated by Gower and colleagues,^7^ whose patient with PPFIBP1-ALK fusion responded to both alectinib and lorlatinib. While we cannot definitively attribute post-progression survival to lorlatinib given subsequent combination therapy, the strategy of next-generation ALK inhibition following first-generation tyrosine kinase inhibitor failure appears rational in ALK fusion-positive PDAC, mirroring the established paradigm in lung cancer.^10^

### Clinical implications

This case reinforces several principles for precision oncology in pancreatic cancer:

1. **KRAS-wildtype PDAC warrants comprehensive genomic profiling.** Our patient’s tumor would have been missed by limited hotspot panels. Broad next-generation sequencing including fusion detection is essential to identify rare actionable events.^11^
2. **ALK fusions, though rare, are highly actionable in PDAC.** The durable response observed here, together with accumulating case reports, suggests that ALK fusions represent a bona fide therapeutic target in this disease.
3. **Response durability can exceed historical expectations.** The 19-month PFS achieved in this case indicates that select patients may derive prolonged benefit from targeted therapy.
4. **Sequential ALK inhibition is feasible.** Our experience supports consideration of next-generation ALK inhibitors upon progression.

### Limitations

This is a single case report with inherent limitations. We cannot exclude that the favorable outcome reflects favorable tumor biology independent of ALK inhibition, though the temporal relationship between alectinib initiation and tumor marker normalization strongly suggests a treatment effect. Additionally, molecular mechanisms of acquired resistance were not assessed at progression.

## Conclusion

We report the first case of ITSN1-ALK fusion in pancreatic ductal adenocarcinoma, demonstrating durable response to alectinib with 19 months of progression-free survival. This case expands the repertoire of ALK fusion partners in pancreatic cancer and adds to the growing body of evidence that ALK inhibition can produce clinically meaningful benefit in appropriately selected patients. Our findings underscore the importance of comprehensive genomic profiling in KRAS-wildtype PDAC to identify rare but actionable driver events.^11^

## Data Availability

All data generated or analyzed during this study are included in this published article. The next-generation sequencing data that support the findings of this case report are available from the corresponding author upon reasonable request, subject to patient privacy protections.
